# Structure-guided mutagenesis of active site residues in the dengue virus two-component protease NS2B-NS3

**DOI:** 10.1186/1423-0127-17-68

**Published:** 2010-08-24

**Authors:** Wanisa Salaemae, Muhammad Junaid, Chanan Angsuthanasombat, Gerd Katzenmeier

**Affiliations:** 1 Laboratory of Molecular Virology, Institute of Molecular Biosciences, Mahidol University, Phutthamonthon 4 Rd., Nakornpathom 73170, Thailand; 2 Department of Pharmaceutical Biosciences, Division of Pharmacology, Uppsala University, 75124 Uppsala, Sweden

## Abstract

**Background:**

The dengue virus two-component protease NS2B/NS3 mediates processing of the viral polyprotein precursor and is therefore an important determinant of virus replication. The enzyme is now intensively studied with a view to the structure-based development of antiviral inhibitors. Although 3-dimensional structures have now been elucidated for a number of flaviviral proteases, enzyme-substrate interactions are characterized only to a limited extend. The high selectivity of the dengue virus protease for the polyprotein precursor offers the distinct advantage of designing inhibitors with exquisite specificity for the viral enzyme. To identify important determinants of substrate binding and catalysis in the active site of the dengue virus NS3 protease, nine residues, L115, D129, G133, T134, Y150, G151, N152, S163 and I165, located within the S1 and S2 pockets of the enzyme were targeted by alanine substitution mutagenesis and effects on enzyme activity were fluorometrically assayed.

**Methods:**

Alanine substitutions were introduced by site-directed mutagenesis at residues L115, D129, G133, T134, Y150, G151, N152, S163 and I165 and recombinant proteins were purified from overexpressing *E. coli*. Effects of these substitutions on enzymatic activity of the NS3 protease were assayed by fluorescence release from the synthetic model substrate GRR-amc and kinetic parameters *K*_m_, *k*_cat _and *k*_cat_/*K*_m _were determined.

**Results:**

Kinetic data for mutant derivatives in the active site of the dengue virus NS3 protease were essentially in agreement with a functional role of the selected residues for substrate binding and/or catalysis. Only the L115A mutant displayed activity comparable to the wild-type enzyme, whereas mutation of residues Y150 and G151 to alanine completely abrogated enzyme activity. A G133A mutant had an approximately 10-fold reduced catalytic efficiency thus suggesting a critical role for this residue seemingly as part of the oxyanion binding hole.

**Conclusions:**

Kinetic data obtained for mutants in the NS3 protease have confirmed predictions for the conformation of the active site S1 and S2 pockets based on earlier observations. The data presented herein will be useful to further explore structure-activity relationships of the flaviviral proteases important for the structure-guided design of novel antiviral therapeutics.

## Background

Dengue virus, a member of the *Flaviviridae *family, is a small, spherical, enveloped, positive single strand RNA virus that is transmitted to humans by mosquitoes of the species *Stegomyia aegypti *(formerly *Aedes*). All 4 serotypes of the virus (DEN-1, 2, 3 and 4) can cause a spectrum of clinical symptoms including mild dengue fever (DF) and more severe forms of dengue hemorrhagic fever (DHF) and dengue shock syndrome (DSS) [1,2]. An increase of geographical spread, incidence and severity of diseases over the past decade has now stimulated intensive efforts to develop effective antiviral therapeutics which are eventually useful for the prevention and cure of dengue virus infections. The development of small molecule drugs directed at inhibition of replication and maturation of the virus is now considered as promising route for the treatment of acute dengue diseases [*for review see *[3-5]*and references herein*].

The dengue virus NS3 protease, a member of the flavivirin enzyme family (EC 3.4.21.91), is located in the N-terminal 184 residues of the multifunctional 69 kDa NS3 protein and contains a functional catalytic triad consisting of H51, D75 and S135 (in DEN-2) [6]. In addition to the serine protease, the NS3 protein contains enzymatic activities of a nucleoside triphosphatase, a 5' - RNA triphosphatase (RTPase) and a RNA - stimulated RNA helicase [7,8]. The NS3 protease catalyses the post-translational cleavage of the viral polyprotein precursor in the non-structural region at the NS2A/NS2B, NS2B/NS3, NS3/NS4A and NS4B/NS5 sites and at additional sites within the viral capsid protein, NS2A, NS4A and within a C-terminal region of NS3 itself [9-13]. The overall conformation of the dengue virus NS3 protease displays the β-barrel conformation typical for serine proteases, although the viral enzyme appears to possess higher compactness with short or absent loop structures and a relatively shallow substrate binding site [14].

The presence of a small hydrophilic core segment of approximately 40 residues, commonly designated NS2B(H), within the small 14 kDa NS2B cofactor is required for optimal activity of the NS3 protease [15-17]. Proteolytic autoprocessing at the NS2B/NS3 site generates a non-covalent adduct between NS2B(H) and NS3 which is catalytically active with substrates supplied in *trans *cleavage reactions [18].

Detailed substrate specificity studies have established that the cleavage junctions in the viral polyprotein consist of pairs of dibasic amino acids such as RR, RK and KR at the P1 and P2 positions. Small, non-branched amino acids such as S are preferred at the P1' position of the dengue virus cleavage site, whereas the preferred P1' residue of the WNV NS3 protease is G [19-21].

Theoretical molecular interactions between the active site of the NS3 protease and the peptide substrate were largely consistent with data obtained from substrate profiling studies [22]. Crystallographic studies of flaviviral proteases including the West Nile Virus (WNV) and dengue virus in complex with a partial NS2B cofactor and substrate-mimetic inhibitors such as aprotonin have provided evidence for major structural reorganizations of the active site pockets caused by insertion of a β-barrel of the NS2B cofactor and an "induced fit" mechanism of catalysis in the presence of authentic protein substrates [23]. Based on a homology-modelled structure of the WNV NS3 protease, residues within the S1 and S2 pockets critical for enzyme-substrate interaction were identified by analysis of catalytic activity of mutant proteases with a synthetic peptide substrate [24]. Structural data obtained recently for a WNV NS2B-NS3pro protease in complex with a substrate-based tripeptide inhibitor have revealed a catalytically competent oxyanion binding site formed by two residues, G133 and S135, and substitution of the active-site nucleophile serine by alanine does not result in a disruption of the oxyanion conformation [25]. It is noteworthy that also in the presence of ligands without a P1' residue the active conformation of the oxyanion hole is adopted by the viral protease.

A high conservation of sequences within the faviviral proteases suggests that specificity characteristics found for the WNV protease could also be of relevance for the closely related dengue virus NS3 protease. Despite their overall similarities, the NS3 proteases from dengue virus and WNV exhibit different substrate specificities, suggesting a distinct organization of their respective active site conformations [21].

In analogy to procedures previously described for the enzyme from WNV, we have identified key residues for substrate binding and catalysis of the dengue virus NS3 protease by alanine substitution mutagenesis and assay of the recombinant mutant enzymes with a synthetic model substrate. In fact, an earlier study has described extensive mutagenesis within the dengue virus NS3 protease for ultraconserved residues among flaviviral proteases and these residues were putatively involved in catalysis or substrate binding [26]. However, activity of the mutant proteases was assayed by SDS-PAGE analysis of autoproteolytic cleavage of the NS2B-NS3 precursor *in vivo*. Although this approach yielded semiquantitative data for activity of the mutant enzymes, it did not provide precise numerical values for the kinetic activity of the mutant proteases with substrates supplied for *trans *cleavage reactions. Moreover, a number of residues such as L115, S163 and I165 have not been included in that investigation as their possible role for enzyme activity was suggested later by data from structural experiments [14,23]. Therefore, the changes in catalytic efficiency which we have observed in the context of amino acid exchanges could contribute to a refined model of substrate specificity and active site conformation for the dengue virus NS3 protease.

## Methods

### Construction of active site mutants of the dengue virus NS2B(H)-NS3pro protease

Plasmid DNA encoding the NS2B(H)-NS3pro precursor of dengue virus serotype 2 strain 16681 cloned in the pTrcHisA expression vector (Invitrogen) containing residues 48-95 followed by residues 121-130 and the N-terminal 180 amino acids of the NS3 protein was used as template for site-directed mutagenesis by using the QuickChange site-directed mutagenesis kit (Stratagene) as described earlier [15]. All synthetic oligonucleotides were purchased from Proligo Pty., Singapore. Mutagenic primers were designed to induce alanine substitutions at positions L115, D129, G133, T134, Y150, G151, N152, S163 and I165 within the NS3pro protein. PCR reactions were carried out by using an automated thermal cycler (Perkin Elmer). Additional restriction sites were incorporated in the primer sequence for screening purposes. A catalytically inactive S135A mutant of NS3pro was used as negative control. Plasmid DNA obtained from recombinant clones was subjected to DNA sequence analysis by using an ABI PRISM™Dye Terminator Cycle Sequencing kit on a model 377 DNA sequencer (Perkin-Elmer, Norwalk, USA). No mutations were found to be present in the plasmid samples at non-targeted sites.

### Expression and purification of the mutant NS2B(H)-NS3pro proteases

Plasmid DNA containing recombinant sequences of the dengue virus NS3pro mutants was transformed in *E. coli *C41(DE3) host cells and cultures were incubated at 37°C in the presence of 0.1 mM IPTG for 8 hours. Protein complexes were purified from inclusion bodies by a two-step procedure using a Hitrap chelating column (Pharmacia) and a Superdex 75 HR 10/300 gel filtration column (Pharmacia) as described earlier [20]. Purification was performed under denaturing conditions in the presence of 8 M urea and purified proteins were refolded by step-wise dialysis using Spectra/Por 6 regenerated cellulose dialysis membranes (Spectrum Laboratories). Samples were concentrated by using Amicon Ultra-15 centrifugal filter devices (Millipore). Protein concentrations were determined by using a BioRad protein assay dye reagent kit based on the Bradford method with bovine serum album (Sigma Chemistry) as standard. Protein samples were analyzed on 15% SDS-PAGE gels using a Mini-Protein III electrophoresis system (BioRad). Preparations of the refolded enzymes were stored at -20°C in 0.1 M Tris-HCl, pH 9.0, 50% (v/v) glycerol for up to 1 week.

### Assay of enzymatic activity

Enzymatic activity of the NS2B(H)-NS3pro recombinant proteases was assayed with the fluorogenic peptide substrate, tBoc-Gly-Arg-Arg-4-methylcoumaryl-7-amide (Peptides International), in 50 mM Tris-HCl, pH 9.0, 20% (v/v) glycerol, by using an automated microtiter plate fluorescence reader (Perkin Elmer) at excitation wavelength λ = 355 nm and emission wavelength λ = 460 nm as described previously [20]. Briefly, reactions were initiated by mixing the substrate solution with the enzyme and fluorescence signals were recorded at 5 minutes intervals over a period of 20 min at 37°C. Initial velocities were corrected for inner filter effects as described in the literature [27]. Concentrations of proteases used in the assay were dependent on activity and were varied between 0.15 μM and 3.0 μM, substrate concentrations were in the range between 12.5 μM and 1.0 mM. Fluorescence signals were converted to product formation by comparison with standard amounts of amc (Sigma Chemistry). Kinetic parameters, *K*_m _and *V*_max_, were obtained from measurement of corrected velocities and Michaelis-Menten kinetics, v = *V*_max _[S]/[S]+*K*_m_, were transformed into double-reciprocal Lineweaver-Burk plots by non-linear regression analysis using the GraphPad Prism 4 software. Three independent experiments were carried out for each set of data points and data are reported as mean ± SEM by one-way analysis of variance calculated by using GraphPad InStat 3 software. Standard deviations of the reported numerical values were < 10%.

## Results and discussion

### Construction, expression and purification of active site mutants in the dengue virus NS3 protease

**Figure 1:**
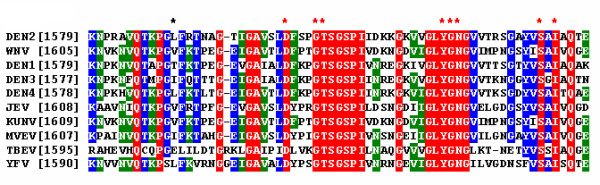
**Multiple alignment of amino acid sequences in the active site region of flaviviral NS3 proteases**. Numbers on the left indicate the startpoint of the amino acid sequence in the viral polyprotein. The degree of conservation among the 10 sequences is represented by background shading of the residues with red, blue, and green shading for 100%, 80-90%, and 60-70% residue conservation, respectively. Residues within the dengue virus serotype 2 sequence substituted by alanine are labeled with asterisks. **Abbreviations: **DEN 1, 2, 3, 4, Dengue Virus serotypes 1, 2, 3, 4 respectively; WNV, West Nile Virus; JEV, Japanese Encephalitis Virus; KUNV, Kunjin Virus; MVE, Murray Valley Encephalitis Virus; YFV, Yellow Fever Virus; TBEV, Tick-Borne Encephalitis Virus.

**Figure 2:**
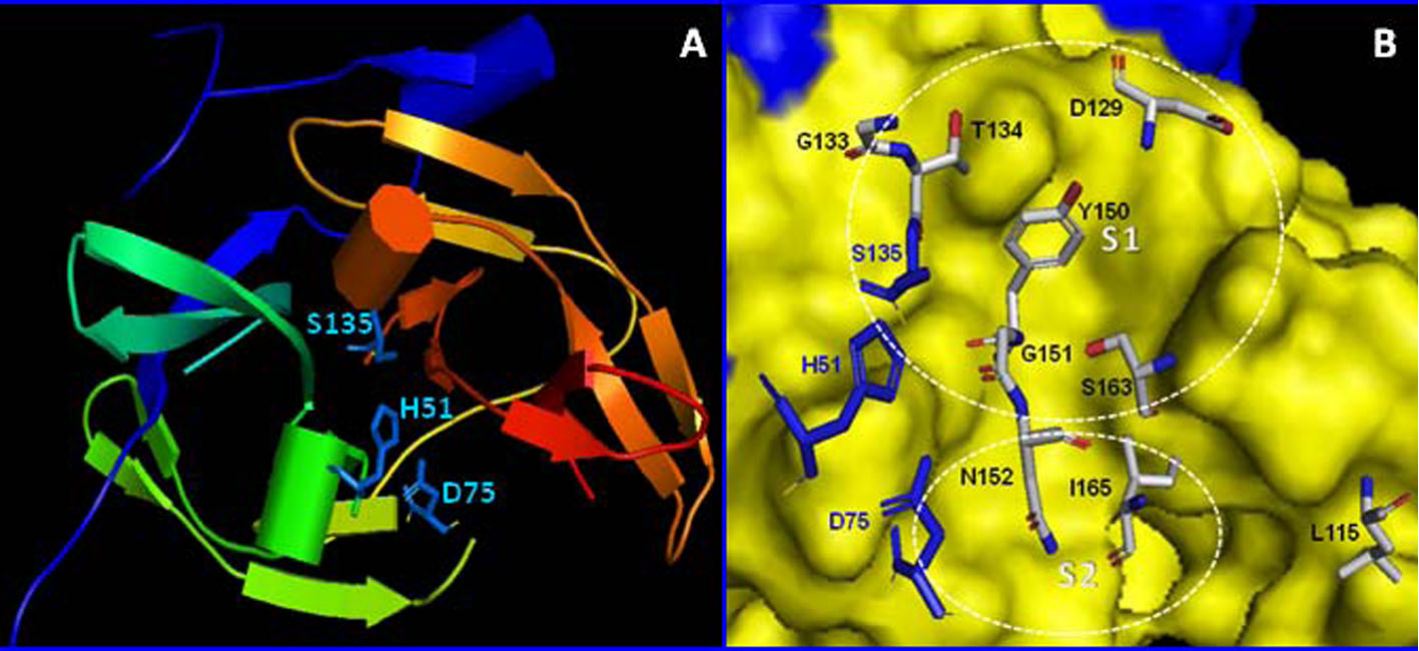
**Structure of the dengue virus NS3 protease active site**. ***Panel A***: Overall structure of the dengue virus NS3 serine protease in complex with a partially resolved structure of the NS2B cofactor domain (in blue). Residues of the catalytic triad H51, D75 and S135 are shown as blue-colored stick models. Data were obtained from the protein data bank at accession code 2FOM (14). ***Panel B***: Zoom-in view of the NS3 protease active site. Residues located within the S1 and S2 binding pockets that were targeted by alanine substitution mutagenesis are shown as element-coloured stick model, labeled in black. Residues labeled in blue represent the members of the catalytic triad H51, D75 and S135.

The NS3 proteases of human-pathogenic flaviviruses such as dengue virus and West Nile virus have received substantial scientific attention as potential targets for the development of antiviral therapeutics. The exquisite selectivity of these proteases for their corresponding polyprotein substrates can be explained by the existence of specific binding pockets for amino acid side chains of the substrate [28]. The aim of our study was a better understanding of structural determinants of substrate specificity of the dengue virus NS3 protease. To this purpose, we have generated alanine substitutions at selected positions within the active site, overexpressed, biochemically purified and assayed enzymatic activity of the recombinant proteins with the synthetic model substrate GRR-amc as described earlier in the literature [15]. The selection of residues potentially involved in enzyme-substrate interactions was based on previous reports for the closely related West Nile virus NS3 protease and predictions extracted from 3-dimensional structures of the dengue virus NS3pro [14,24,25]. Residues L115, D129, Y150 and S163 were predicted to line the S1 subsite of the protease, while N152 was proposed to present a key residue in the S2 subsite. With the exception of position 115, all these residues are strictly conserved among all flaviviruses. An alignment of active site residues for a number of flaviviral proteases is shown in Fig. 1. A structural analysis of the WNV NS3 protease recently published by Robin *et al. *has suggested a role for residue G133 in the formation of the oxyanion hole [25]. A map of the dengue virus NS3 protease active site with residues putatively involved in enzyme-substrate interaction is shown in Fig. 2[14]. Alanine mutations were introduced in the NS3 protease at residues L115, D129, G133, T134, Y150, G151, N152, S163 and I165 by using the previously described construct NS2B(H)-NS3pro as a template for site-directed mutagenesis by PCR [16].

**Figure 3:**
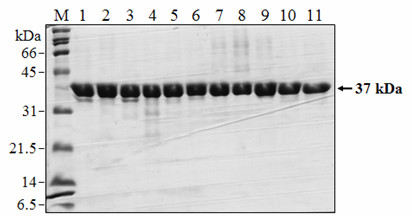
**SDS-PAGE analysis of purified proteases NS2B(H)-NS3pro**. Proteins were obtained by a two-step purification procedure using immobilized metal affinity chromatography and size-exclusion chromatography on a Superdex 75 HR10/300 GL column as described under Methods. Proteins shown were loaded in the presence of 8 M urea under denaturing conditions prior to refolding. Lane M, molecular weight marker proteins with molecular weight indicated; lane 1, NS2B(H)-NS3pro protease wild-type; lane 2, inactive S135A mutant of NS3pro; lanes 3-11, purified protein samples of nine active site mutants L115A, D129A, G133A, T134A, Y150A, G151A, N152A, S163A and I165A in NS2B(H)-NS3pro, respectively. The arrow indicates the band of NS2B(H)-NS3pro migrating at an apparent molecular weight of 37 kDa. Samples were run on 15% SDS-PAGE gels and stained with Coomassie Brillant Blue.

All wild-type and mutant proteins were expressed as insoluble inclusion bodies and a two-step purification under denaturing conditions by immobilized metal affinity chromatography and size-exclusion chromatography resulted in greater than 95% pure proteins as judged by SDS-PAGE (Fig. 3). Refolding of the samples by step-wise dialysis yielded enzymatically active proteins as described earlier [20].

Analysis of autoproteolysis as described earlier for mutants in the NS2B(H) activation sequence [15] revealed that the Y150A and G151A mutants were completely inactive with no products of self-cleavage detectable (data not shown).

### Kinetic analysis of active-site mutations

**Figure 4:**
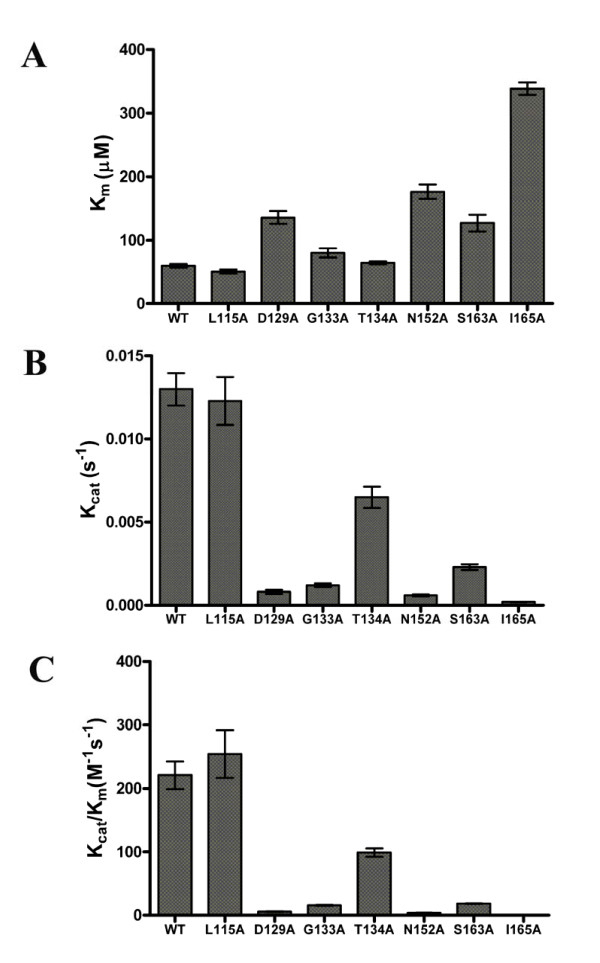
**Presentation of kinetic parameters for samples of NS2B(H)-NS3pro wild-type and mutant derivatives**. Samples were assayed by using fluorescence emission from cleavage of the peptide substrate GRR-amc at 37°C as described under Methods. The bar graph shows a comparison of numerical constants obtained for *K*_m _(***panel A***), *k*_cat _(***panel B***) and catalytic efficiency *k*_cat_/*K*_m _(***panel C***) for the wild-type protein NS2B(H)-NS3pro and active site mutant proteins L115A, D129A, G133A, T134A, N152A, S163A and I165A. Samples of Y150A and G151A were inactive in the enzyme assay and a 23-fold increase in enzyme concentration did not result in detectable activity. Data represent the mean of triplicate measurements and error bars are indicated.

We have assayed the enzymatic activity of the mutant enzymes by using the synthetic substrate tripeptide GRR with a conjugated fluorescence reporter group, amc, with inner filter effect correction as described earlier [27]. Recombinant NS2B(H)-NS3pro proteases were assayed for activity at various concentrations of protein and substrate as described in Materials and Methods and kinetic parameters, *K*_m _and *k*_cat_, were calculated from Michaelis-Menten kinetics. The alanine mutations introduced at selected residues within the active site of the NS3 protease had marked effects on substrate binding (*K*_m_) and rates of substrate hydrolysis (*k*_cat_) when compared to the activity of the wild-type enzyme (Fig. 4). As shown by comparison of *K*_m _values, all mutations except the L115A mutant resulted in effects on substrate affinity and increased *K*_m _values, whereas catalytic rates *k*_cat _appeared to be significantly reduced for all mutants except L115A. The most notable effect was observed for the Y150A and G151A mutants that displayed negligible activity under the conditions of the assay and a 23-fold increase in enzyme concentration over the amount used for the wild-type protein did not result in detectable activity, thereby suggesting that these mutations completely inactivate the enzyme. The changes in catalytic efficiency observed for the mutant NS3 enzymes can therefore be summarized in the order:

L115A>wild-type>T134A>S163A>G133A>D129A>N152A>I165A.

#### G151 and G133

Contrary to our expectations, alanine substitution of G151 yielded an enzyme with completely abolished activity. G151 is invariantly present in the NS3 sequences of known flaviviruses and we speculated that this residue might play a dual role in the stabilization of the tetrahedral transition state intermediate formed at S135 during substrate cleavage and to maintain structural stability of the E2-F2 strands in the protease fold [14]. G133 is part of the ultraconserved GxSGxP motif found in flaviviral NS3 sequences and most likely determines the optimal size and generates the catalytically competent conformation of the oxyanion hole for the accommodation of the respective substrate [25]. Earlier reports have proposed that substrate-free conformations of the NS2B-NS3pro enzyme contain a flipped peptide bond between T132 and G133 which abrogates the active conformation of the oxyanion hole and that formation of the active oxyanion hole by an induced fit mechanism requires the presence of authentic substrates containing a P1' residue [14,23]. However, it was demonstrated recently that the active conformation of the oxyanion hole in WNV NS3 protease is maintained also in the presence of inhibitors without a P1' residue [25].

Alanine substitution of the G133 residue resulted in a NS3 protease with only approximately 10-fold reduced catalytic efficiency when compared with the wild-type enzyme, thus suggesting some degree of conformational freedom at this position.

#### Y150

Substitution of Y150 by alanine yielded a NS3 protease with completely abrogated activity, a finding in agreement with earlier mutagenesis data for this residue from WNV and dengue virus [24,25]. It was suggested that Y150 could primarily stabilize the positively charged side chain of the P1 arginine by an aromatic π - cation interaction. An additional role for Y150 could be the structural stabilization of the E2 strand in the C-terminal β-barrel of the NS3 protein.

#### S163

This position is structurally homologous to residue 226 of chymotrypsin which is part of the portal to the substrate binding pocket and is conserved in all flavivirus proteases. It was proposed earlier that G153 and S163 form a bulkier entry to the substrate binding pocket [26]. Mutation of S163 to alanine in the WNV NS3 protease generated an inactive enzyme [24], whereas we have found a 12-fold reduction in catalytic efficiency for the dengue virus S163A mutant. This residue could play a role in substrate binding by formation of a hydrogen bond with the substrate P1 arginine.

#### D129

Substitution of the invariant residue D129 by alanine induced a large (39-fold) decrease in catalytic efficiency. Chappell *et al. *have reported a 17-fold increase in *K*_m _for the D129A mutant of the WNV protease, whereas we have seen a 2.3-fold increase in *K*_m _and a 16-fold decrease in *k*_cat _[24]. The precise role of this residue requires further experimental analysis, however, the possibility exists that D129 participates both in substrate binding by providing salt bridge or hydrogen bond interactions with the substrate P1 arginine as well as in the catalytic mechanism of the NS3 protease.

#### T134

Alanine substitutions of this residue had only minor effects on enzymatic activity as demonstrated by a 2.2-fold decrease in catalytic efficiency. T134 could provide a weak interaction with the P1 arginine, presumably via a hydrogen bond from its hydroxyl group.

#### L115

A V115F mutant of the WNV NS3 protease was enzymatically inactive most likely due to size restrictions which prevented the substrate from occupying the S1 pocket [24], however, in the dengue virus NS3 protease L115 appears to be located at a position relatively remote from the S1 pocket (Fig. 2) [14]. For the L115A mutant of dengue virus NS3 protease, we have observed even a marginal increase in catalytic efficiency suggesting that a smaller side chain could provide additional flexibility for the accommodation of the P1 substrate.

#### N152

In contrast to findings with the WNV virus NS3 protease, the N152A mutation did not completely inactivate the dengue virus enzyme but resulted in a substantial 60-fold reduction of catalytic efficiency. N152 is part of the S2 subsite and presumably provides an interaction with the side chain of the P2 substrate via hydrogen bonding [14].

#### I165

The role of this residue was not investigated in previous studies although it is conserved throughout the flavivirus NS3 proteases and was predicted to line the S1 pocket. Mutation of this residue to alanine removes the bulky side chain and results in a drastic increase in *K*_m _thus suggesting a function in substrate binding rather than catalysis, conceivably by a reduction of the size of the S1 pocket.

Taken together, these results have largely confirmed previous predictions for active site residues of the dengue virus NS3 protease and therefore suggest that the residues selected in this study have probably a major function for substrate binding and catalysis. In addition to existing structural data we have further obtained enzymatic evidence to suggest that the residues G133 and G151 play a role in the catalytic mechanism of the dengue virus NS3 protease, presumably as structural elements of the oxyanion binding hole. Although our findings for the dengue virus NS3 protease are essentially in agreement with previous data for the WNV NS3 protease, we have observed that a number of residues such as S163, N152 and I165 display distinct effects on enzyme activity when substituted by alanine. These differences could relate to subtle structural alterations in the structures of the WNV and dengue virus proteases [29]. Moreover, differences in substrate specificity were recently explained by alteration of the substrate binding pockets in the S2-S4 region by dissimilarities in NS2B complexation between the dengue virus and WNV NS3 proteases [30]. It is also noteworthy that the S1 and S2 pockets of a proteolytic enzyme may not be the sole determinants of specificity [31]. Nevertheless we believe that the S1 and S2 residues identified in this study may represent ideal targets for the design of antiviral inhibitors against NS3 serine proteases of dengue virus and possibly other flaviviruses.

## Conclusions

The design of inhibitors against the flaviviral NS3 serine proteases requires a precise knowledge of the structural determinants of substrate binding and catalysis. In this study we have re-visited the conformational properties of the dengue virus NS3 protease active site by a structure-guided mutagenesis approach of nine residues located within the S1 and S2 binding pockets. Of these, all with exception of L115 had prominent effects on enzyme catalysis and therefore represent important functional determinants for substrate specificity. To the best of our knowledge, this study describes for the first time a kinetic analysis of mutations in the dengue virus NS3 protease by a *trans *cleavage assay with a synthetic peptide model substrate.

These structural requirements can be utilized in informed drug discovery programs aiming at the discovery of selective inhibitors against the flaviviral NS3 proteases.

## Abbreviations

DEN: dengue virus; DEN-2: dengue virus serotype 2; DF: dengue fever; DHF: dengue hemorrhagic fever; DSS: dengue shock syndrome; GRR-amc: tBoc-Gly-Arg-Arg-4-methylcoumaryl-7-amide; NS: non-structural; NS2B and NS3: dengue virus non-structural viral proteins 2B and 3: respectively; NS3pro: the protease domain of the NS3 protein; NS2B(H): the central hydrophilic activation domain of the NS2B protein; PAGE: polyacrylamide gel electrophoresis; WNV: West Nile virus.

## Competing interests

The authors declare that they have no competing interests.

## Authors' contributions

WS constructed the active site mutants of the dengue virus NS3 protease, purified the recombinant proteins and assayed their enzymatic activities. MJ participated in the analysis and interpretation of the data by computational methods. CA was involved in study design and coordination of experimental work. GK has conceived the study and wrote the manuscript. All authors read and approved the final manuscript.
